# The effect of pre-cure bracket movement on shear bond strength during placement of orthodontic brackets, an *in vitro* study

**DOI:** 10.4317/jced.54208

**Published:** 2017-10-01

**Authors:** Byron Tam, Prashanti Bollu, Kishore Chaudhry, Karthikeyan Subramani

**Affiliations:** 1 Roseman University of Health Sciences, College of Dental Medicine, Henderson, NV, USA

## Abstract

**Background:**

The purpose of this study was to determine the influence of linear and rotational pre-cure bracket displacement during the bonding procedure on shear bond strength (SBS) of orthodontic brackets.

**Material and Methods:**

Stainless steel orthodontic premolar brackets were bonded to the buccal surfaces of 50 human pre-molars with a conventional two-step bonding protocol. Extracted human pre-molars were divided into 5 groups (n=10/group). In the Control Group, the brackets were bonded with no pre-cure bracket displacement or rotation. The Rotation Group was bonded with 45 degrees of pre-cure rotation. The Displacement Group was bonded with 2mm pre-cure linear displacement. The Rotation-Displacement Group was bonded with pre-cure movements of 45º counter-clockwise rotation and 2mm displacement. The Slippage Group was bonded with 2mm each of mesial and distal pre-cure linear displacement. Photo-activation was carried out on the lateral sides of the bracket. Shear debonding force was measured, 24 hours after initial bonding, with an Instron universal testing machine using a knife-edged chisel. Data was analyzed using one-way ANOVA test. Adhesive Remnant Index (ARI) was scored under 15x magnification. The ARI data was analyzed using the Chi-square test (*p* -value < 0.05)

**Results:**

No statistically significant differences were detected among the control and experimental groups (*p* = 0.331). The rotation and displacement group showed the highest mean SBS than all other groups. Mean SBS for all groups were above the clinically acceptable range. No statistically significant differences were detected in ARI scores among groups (*p* = 0.071).

**Conclusions:**

Linear and rotational pre-cure bracket displacements do not appear to effect the shear bond strength of orthodontic brackets.

** Key words:**Shear bond strength, orthodontic bracket, displacement, rotation, adhesive remnant index, pre-cure movement.

## Introduction

The concept of bonding dental materials to enamel was first introduced by Buonocore (1) in 1955. This was then applied to orthodontics when Newman (2) and Retief (3) modified the phosphoric acid concentration and application time in order to bond orthodontic attachments to enamel by using epoxy resins. By 1972, patients were being successfully treated with orthodontic brackets bonded to enamel (4). Developments in dental materials and adhesives over the last 45 years have made the direct bonding technique a routine part of fixed orthodontic treatment.

In addition to dental adhesives, orthodontic bracket design has undergone major improvements in recent decades, most notably the pre-adjusted orthodontic appliance introduced by Andrews (5). The primary source of movement (torque and angulation) was designed into the bracket allowing the clinician to obtain a high degree of tooth control and improved the functional positioning of teeth by reducing the amount of archwire manipulation. However, Andrews noted that the successful use of pre-adjusted appliance required accurate bracket placement, as they were designed for placement at an ideal position on the crown of the tooth (5). Thus, the clinician must have the ability to consistently and accurately identify certain anatomical landmarks and visualize angular and linear features of the crown. Bracket placement has been described as the most important step and incorrect bracket placement may necessitate several additional months of treatment to finish an individual case (6).

When clinicians attempt to locate the ideal position to place and bond a bracket, manipulation of the bracket on the tooth surface is often necessary. The need for displacement and rotation of the bracket may be increased with posterior teeth due to poor accessibility caused by the oral musculature. Excessive bracket movement during the bonding procedure could displace the adhesive resin from the bracket pad and interfere with the bracket-enamel interface bond. There are no studies done to evaluate the effect of bracket movement on Shear Bond Strength (SBS).

The success of fixed appliance therapy depends on the bracket having adequate bond strength to enamel. Ideal SBS has been determined to be approximately 5.8-7.9 MPa, which should be sufficient to withstand bracket displacement and resist tensile, shear, torque and functional stresses due to intraoral forces during the course of treatment (7). Bond failures, however, are frequently encountered during orthodontic treatment and have been found to occur at an average rate of 6-17.6% (8-11). Bond strength may be influenced by factors such as etchant concentration, etching time, bonding agent type, or bracket base characteristics (9,10). Bracket failures can lead to loss of already achieved tooth movement and thereby increasing overall treatment time. Achieving a low bracket failure rate should be a high priority objective because replacing brackets is inefficient, time-consuming and costly for the clinician besides causing inconvenience to the patient.

It is uncertain whether the movement of an orthodontic bracket on the tooth surface prior to photo-activation of the adhesive material has an effect on bond strength. The purpose of this study was to investigate if linear and rotational pre-cure orthodontic bracket displacement during the bonding procedure influenced SBS.

## Material and Methods

-Test Samples

Fifty extracted human mandibular premolars with intact buccal surfaces were collected. Teeth were stored in a 1:100 sodium hypochlorite (Clorox, Oakland, CA) solution from the time of collection to the time of bonding. Teeth were mounted in 0.5 inch diameter PVC Sch 40 Plug (Lasco, Brownsville, TN) using Type III Dental Stone (GC America Inc., Alsip, IL) with the buccal surface perpendicular to the horizontal plane.

-Exclusion Criteria

Teeth with hypoplastic areas, cracks, gross irregularities in enamel structure, caries, and stains (extrinsic) were excluded from the study.

-Brackets and Bonding Materials

Fifty American Orthodontics Mini Master SeriesTM - Mandibular Right 1st premolar metal brackets (Sheboygan, WI) with a bracket base area of 13.6 mm² were used in this study. The etchant used was 35% phosphoric acid Opal Etch (Ultradent, South Jor-dan, UT). Assure 6cc Bonding Resin primer (Reliance Orthodontic Products, Inc., Itasca, IL) and Assure Light Bond adhesive (Reliance Orthodontic Products, Inc., Itasca, IL) were used.

-Bonding Procedure

1. Each tooth received 10 seconds of prophylactic treatment with non-fluoride containing pumice (Henry Schein, Melville, NY), water rinse for 10 seconds, and dried with pressurized air for 5 seconds.

2. Opal® etchTM (35% phosphoric acid) was applied to enamel and left in place for 30 seconds, then rinsed thoroughly for 20 seconds.

3. A thin layer of Assure 6cc Bonding Resin was applied to the etched enamel and air dried for 2 seconds according to manufacturer’s recommendation. Tack-cure Assure 6cc Bonding Resin was used for 5 seconds to avoid “skating” upon bracket placement.

4. A thin layer of Assure Light Bond adhesive was applied to bonding surface of an American Orthodontics Mini Master SeriesTM - Mandibular Right 1st premolar metal bracket.

5. The orthodontic brackets were placed in a standardized manner with identical pressure applied to each bracket using a force gauge (Dontrix gauge) to approximately 300g.

6. The teeth were divided into five groups (n=10/group), according to the bonding protocol as follows:

Group 1: Control Group: The bracket was positioned on the center of the tooth with 300g force applied with Dontrix gauge (American Orthodontics, Sheboygan, WI) and the archwire slot was perpendicular to long axis of the tooth. Flash was carefully removed and then light cured for 15 seconds each on mesial and distal of the bracket using a light emitting diode curing unit at 1,200 mW/cm² (American Orthodontics, Sheboygan, WI).

**Figure 1:**
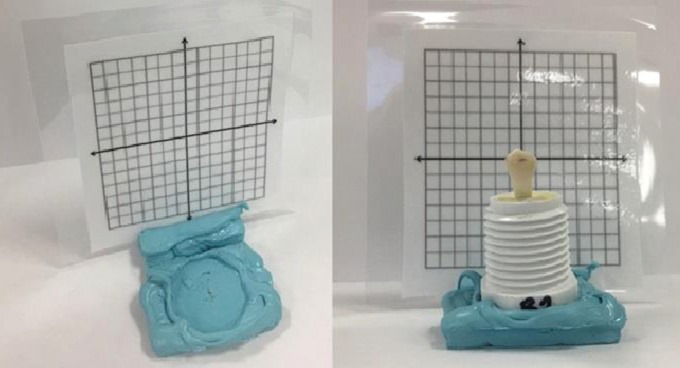
Mounting jig for standardization of 45º rotational movement.

Group 2: Rotation Group: The bracket was positioned on the center of the tooth with the archwire slot 45 degrees counter clock-wise to the tooth long axis measured with a mounting jig (Fig. 1). Uniform force of 300g was applied with a Dontrix gauge. Bracket was then rotated clockwise so that archwire slot is perpendicular to the tooth long axis. Same force of 300g was re-applied with the Dontrix gauge, flash was carefully removed and light cured for 15 seconds each on mesial and distal sides of the bracket.

Group 3: Displacement Group: The bracket was positioned 2mm gingival from the center of the tooth. 300g of force was applied to bracket with Dontrix gauge with the archwire slot perpendicular to long axis of the tooth. The bracket was then displaced 2mm incisally to the center of the tooth. 300g of force was reapplied with Dontrix gauge, flash was carefully removed and bracket was light cured for 15 seconds on both mesial and distal sides.

Group 4: Rotation and Displacement Group: The bracket was positioned 2mm gingival from the center of the tooth with the archwire slot 45 degrees counter clockwise to the tooth long axis and 300g of force was applied with the Dontrix gauge. Bracket was then rotated clockwise so that the archwire slot is perpendicular to long axis of the tooth and then displaced incisally 2mm. 300g of force was reapplied with Dontrix gauge, flash was carefully removed and light cured for 15 seconds on both mesial and distal sides of the bracket.

Group 5: Slippage Group: The bracket positioned on the center of the tooth with 300g force applied with Dontrix gauge and the archwire slot was perpendicular to long axis of the tooth. Flash was carefully removed and the bracket was displaced distally 2mm, then mesially 2mm. 300g of force was reapplied with Dontrix gauge and light cured for 15 seconds on each mesial and distal of the bracket.

The same operator carried out all bonding procedures.

-Testing Procedure

**Figure 2:**
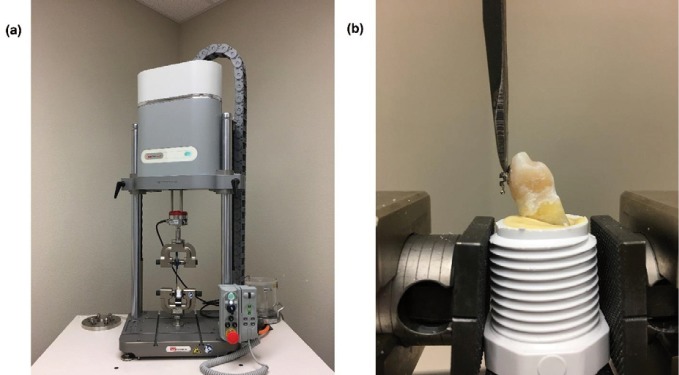
(a) Instron testing machine with sample held in position. (b) Instron attachment blade placed at the bracket ligature groove ready for testing at a crosshead speed of 1mm/min.

SBS testing was conducted 24 hours after photofixation. The specimen were stored in distilled water at room temperature (23ºC). The teeth were positioned in an Instron Electropuls E1000 Universal testing machine (Instron Corp., Norwood, MA). The archwire slot was parallel to the horizontal plane. A knife-edged chisel was placed in contact with the incisal portion of the bracket in between the enamel and tie wings (ligature groove) parallel to the long axis of the tooth, creating a shear force in the occluso-gingival direction (Fig. 2). The specimen was subjected to a compressive load at a crosshead speed of 1.0 mm/min until failure. The force in Newtons required to debond each bracket was recorded.

-Adhesive Remnant Index

The debonded brackets were observed under 15x magnification with a macro lens. Photographs were taken of each bracket to score the adhesive remnant index (ARI). ARI were recorded for each bracket at two separate time points two weeks apart. ARI was scored according to the following grading system (12):

0: 100% of the adhesive remaining on the bracket

1: More than 50% of the adhesive remaining on the bracket

2: Less than 50% of the adhesive remaining on the bracket

3: No adhesive remaining on the bracket

-SBS Calculation and Statistical Analysis

SBS was calculated using the following formula:

SBS in Megapascals = Force in Newtons/ Surface area of bracket base in mm2

Mean and standard deviation of SBS values were calculated by using SPSS version 23 (IBM Chicago, IL, USA). One-way ANOVA test was used for multiple comparisons of SBS between groups with *p*-value set at 0.05. The ARI scores were compared using a chi-square analysis to determine if there was a significant difference in mode of bond failure among groups.

## Results

Results for the SBS values are shown in  Table 1. No significant differences were detected among the control and experimental groups (*p* = 0.331). The teeth bonded with rotation and displacement showed the highest SBS of 13.85 ± 4.88 MPa. The lowest SBS was 10.66 ± 3.18 MPa, for the slippage group. The two groups with rotational movements had the highest average SBS, 13.40 ± 5.37 MPa for the rotation only group and 13.85 ± 4.88 MPa for the rotation and displacement group. The two groups with linear displacement only, had the lowest average SBS, 11.13 ± 3.49 MPa for the displacement group and 10.66 ± 3.18 MPa for the slippage group. The SBS of control group was 12.51 ± 2.54 MPa. The rotation only group and rotation and displacement group had the largest SBS measurements, 24.6 MPa and 23.1 MPa respectively. The displacement group and slippage group had the lowest SBS measurements, 6.1 MPa and 6.6 MPa respectively.

**Table 1:**
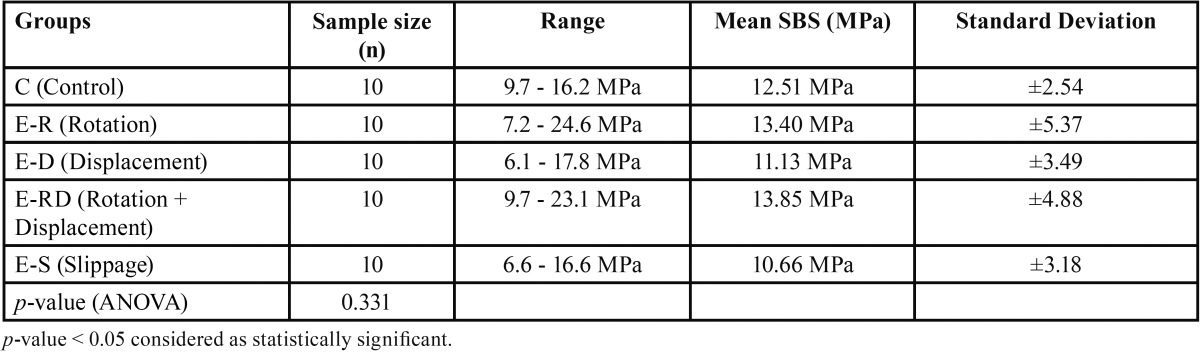
Mean shear bond strength in megapascals (MPa). The results are shown in Mean ± SD.

The results of ARI analysis are shown in  Table 2. No significant differences were detected in mode of bond failure among groups (*p* = 0.162). The group with an ARI score of 1 (having more than 50% of adhesive remaining on the bracket) had the most observations of 24. The group with an ARI score of 3 (no adhesive remaining on the bracket) had the lowest observation of 1. The rotation only group did not experience any brackets with an ARI score of 2 or 3.

**Table 2:**
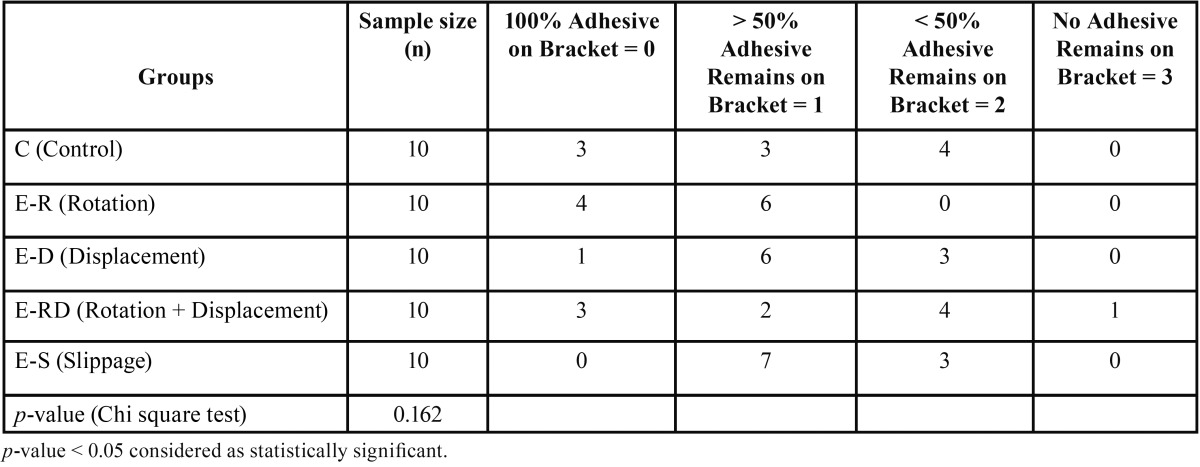
Adhesive Remnant Index.

## Discussion

The control group showed a mean SBS of 12.51 ± 2.54 MPa. Even though it did not have the highest mean SBS, the control group had the most consistent measurements with the lowest standard deviation of 2.54 MPa and with a range of 9.7 – 16.2 MPa. Using similar bonding protocol, the SBS for the control group of this study was found to be higher than those found in Bishara *et al.* (13) and Oonsombat *et al.* (14), 4.6 ± 3.2 MPa and 6.0 ± 3.5 MPa respectively.

Both groups with rotational movements exhibited the highest mean SBS. The group with the 45º rotational pre-cure bracket movement had a mean SBS of 13.40 ± 5.37 MPa, while the group with the 45º rotational and 2mm linear pre-cure bracket movement showed the highest mean SBS of 13.85 ± 4.88 MPa. These findings were consistent with those found by Oliveira *et al.* (15), where both rotational group’s SBS (9.8 ± 2.2 MPa and 10.1 ± 2.0 MPa) were higher than the control group (9.7 ± 1.8 MPa). The premolar orthodontic brackets pads are designed with more curvature when compared to their incisor counterparts due to the anatomy of the buccal surfaces of the teeth. When the premolar bracket was placed 45º from ideal, it was poorly adapted to the buccal surface so adhesive was allowed to remain in the concave portion of the bracket. As the bracket rotates into position, Oliveira *et al.* (15) speculated that this pre-cure rotational movement may have improved the interlocking of the resin with the bracket mesh. As the bracket adapts to the buccal surface, excess adhesive exudes from around the bracket and is called flash. The flash was not removed until the bracket was in final position, so when the bracket was then displaced vertically 2mm, the excess adhesive from the occlusal portion of the bracket may have been forced underneath the bracket as the bracket slid across the surface of the tooth, thus maintaining a sufficient mechanical bond.

Orthodontists routinely delegate initial bracket placement to chairside assistants. The slippage group was created for the instance when assistants are asked to place brackets from second premolar on one side to the second premolar on the other side in an entire dental arch prior to final fixation by the orthodontist. By the time the assistant reaches the final second premolar on the other side, sufficient time may have elapsed for the bracket that was placed on the first second premolar to have slipped distally and/or gingivally due to gravity as the adhesive remained unpolymerized. Although the frequency of this event occurring has not been studied, this type of movement should be categorized as a pre-cure bracket displacement and was shown to have the lowest mean SBS among the five groups in our study at 10.66 ±3.18 MPa. Even though the slippage group had the lowest SBS, it was still above the clinically acceptable range.

*In vitro* testing has inherent limitations as the study results are not necessarily the same as those that would be obtained in the oral environment. Although this study showed that pre-cure bracket rotation and displacement did not affect SBS, other factors may play a role in reducing the SBS *in vivo*. The sample size for each group in this study (n=10) exceeded the estimated number needed to detect a difference among the study groups (n=7) which was determined by power analysis. Although appropriate, a sample size of 10 may be too small to account for the differences in enamel matrix composition between the premolar specimens, which may have an effect on SBS (16). In order to mitigate the differences in enamel composition among each specimen, increasing the sample size would be recommended for future studies.

The results of the present study provide evidence that linear and rotational pre-cure bracket displacements do not affect the SBS of orthodontic brackets when bonded with a conventional two step bonding protocol. Therefore, linear bracket movements up to 2mm in combination with rotational movements up to 45º to correct the position of the orthodontic brackets on teeth can be carried out without jeopardizing the bond strength to enamel, as the mean SBS of all groups were above the clinically acceptable SBS of 5.8-7.9 MPa (7).

## Conclusions

Direct bonding of orthodontic brackets to teeth requires the bracket to be bonded to the center of the clinical crown. In search of this ideal position, the bracket often requires manipulation in multiple linear and angular directions as the bracket is moved on the buccal surface of the tooth. The results of this study indicate that rotational and linear pre-cure bracket movements do not effect the SBS to enamel. Therefore, the accuracy of initial bracket placement may not be as important a factor affecting the shear bond strength of orthodontic brackets as intuitively expected.
